# Analysis on the interactions between the first introns and other introns in mitochondrial ribosomal protein genes

**DOI:** 10.3389/fmicb.2022.1091698

**Published:** 2022-12-08

**Authors:** Ruifang Li, Xinwei Song, Shan Gao, Shiya Peng

**Affiliations:** College of Physics and Electronic Information, Inner Mongolia Normal University, Hohhot, China

**Keywords:** local matched alignment, first intron, optimal matched segments, mitochondrial ribosomal protein genes, interaction

## Abstract

It is realized that the first intron plays a key role in regulating gene expression, and the interactions between the first introns and other introns must be related to the regulation of gene expression. In this paper, the sequences of mitochondrial ribosomal protein genes were selected as the samples, based on the Smith-Waterman method, the optimal matched segments between the first intron and the reverse complementary sequences of other introns of each gene were obtained, and the characteristics of the optimal matched segments were analyzed. The results showed that the lengths and the ranges of length distributions of the optimal matched segments are increased along with the evolution of eukaryotes. For the distributions of the optimal matched segments with different GC contents, the peak values are decreased along with the evolution of eukaryotes, but the corresponding GC content of the peak values are increased along with the evolution of eukaryotes, it means most introns of higher organisms interact with each other though weak bonds binding. By comparing the lengths and matching rates of optimal matched segments with those of siRNA and miRNA, it is found that some optimal matched segments may be related to non-coding RNA with special biological functions, just like siRNA and miRNA, they may play an important role in the process of gene expression and regulation. For the relative position of the optimal matched segments, the peaks of relative position distributions of optimal matched segments are increased during the evolution of eukaryotes, and the positions of the first two peaks exhibit significant conservatism.

## Introduction

An intron sequence is regarded as a kind of non-coding sequence of interrupted gene, and its functions are being discovered. A large number of studies have shown that intron can regulate gene expression as a kind of regulatory element (Palmiter et al., 1991; Li et al., 2015; Abou et al., 2020), for example, the heterologous introns can enhance expression of transgenes in mice (Palmiter et al., 1991). In recent years, it has also been found that some introns can influence many stages of mRNA metabolism, including initial transcription of a gene, editing of pre-mRNA, and nuclear export, translation and decay of the mRNA (Bo et al., 2019). Furthermore, introns contain kinds of non-coding RNA such as microRNA and snoRNA (Mattick and Gagen, 2001), they also participate in the functional activities of a variety of non-coding RNA (Abou et al., 2020). And it has shown that GC-AG introns are mainly associated with lncRNAs and are preferentially located in the first intron (Abou et al., 2020), additionally, many studies have shown that introns are closely related to various diseases (Sowalsky et al., 2015; Malekkou et al., 2020; Ong and Adusumalli, 2020), for example, a novel mutation deep within intron 7 of the GBA gene can cause Gaucher disease (Malekkou et al., 2020), and increased intron retention is associated with Alzheimer’s disease (Ong and Adusumalli, 2020), and metastatic castration-resistant prostate cancer is related to some non-coding RNA (Sowalsky et al., 2015).

The most basic and important interaction among bases is base matching, for example, the formation of a correct codon-anticodon pair is essential to ensure efficiency and fidelity during translation, and circRNA formed by exon cyclization or intron cyclization contains long flanking introns with complementary repeats (Han et al., 2022). Besides, many studies indicated that intron complementary matching fragments are not only the cause of circular RNA, but also the potential factors for the complexity and diversity of gene expression at the transcriptional/post transcriptional level (Zhang et al., 2013, 2014; Jiao et al., 2021). Therefore, it is particularly important to study the circular matching problem of introns.

The first introns have gained increasing attentions in recent years because of their unique features that are located in close proximity to the transcription, and the distinct deposition of epigenetic marks and nucleosome density on the first intronic DNA sequence (Fu et al., 2022; Singh et al., 2022; Spijker et al., 2022; Vosseberg et al., 2022), and it is realized that the first introns play a key role in several mechanisms regulating gene expression. We determined that the matching features between the first introns and the corresponding reverse complementary sequences of other introns must provide many useful information.

The genome consists of an extremely complex network of interactions among functional elements, and its functions are achieved primarily through these interactions. We have known that a complete match between siRNA and targeted genes can lead to targeted genes silencing, and high but incomplete matching between miRNA and targeted genes can suppress gene expression. It means base matching is an important way for non-coding RNA to interact with targeted genes, intron as a kind of non-coding DNA is rich in eukaryote genomes, introns must interact with each other, and the interactions can be embodied by the modes of base matching. Based on this, in this work, the mitochondrial ribosomal protein gene sequences were selected as samples, the characteristics of the optimal matched segments between the first intron and the corresponding reverse complementary sequences of other introns were analyzed, and the variations of the characteristics along with the evolution of eukaryotes were studied.

## Materials and methods

### Datasets

All the sequences of mitochondrial ribosomal protein genes in the Ribosomal Protein Gene Database (RPG) were selected as our samples, they were from *Homo sapiens, Mus musculus, Fugu rubripes, Drosophila melanogaster* and *Caenorhabditis elegans*. Considering that the mitochondrial ribosomal protein gene has many advantages in biological research as a kind of housekeeping gene, they are involved in the key process of all protein translation and have very good evolutionary conservatism (Yoshihama et al., 2002), thus forming a family of conservative genes. They exist widely in all eukaryotes, and their intron lengths and amounts have little difference in all eukaryotes. We believe that more reliable and functional interactions among introns can be obtained by selecting these conserved genes. Information about the protein genes is given in Table 1.

**Table 1:** Mitochondrial ribosomal protein genes.

Species	The amount of genes	The amount of introns	The amount of first introns
*Homo sapiens*	114	512	114
*Mus musculus*	79	351	79
*Fugu rubripes*	69	266	64
*Drosophila melanogaster*	75	118	66
*Caenorhabditis elegans*	74	251	71

### Matching method

The intron sequences were obtained from the above gene sequences, then, they were transformed into their reverse complementary sequences except the first introns. Next, similar alignments were done by the local similarity matching software called Smith-Waterman.^1^ We adopt Ednafull matrix to similarity matching, and parameters chosen as follows, each Gap penalty is 50.0, in the gap each extend penalty is 5.0, thus, we got the optimal similar segment between the first intron and corresponding reverse complementary sequences of other introns in each gene sequence.

### The optimal matching frequency

We calculated the length, GC content, matching rate of each optimal matched segment considering that they must provide the basic characteristics of the optimal matched segments, then divided the optimal matching segments into several groups, respectively, according to their lengths, GC contents or matching rates. And then calculated the frequencies of the optimal matched segments with different ranges of lengths, GC contents and matching rates, marked with *F*_*L*m_, *F*_GCm_, and *F*_mat_ respectively, they were defined as follows,


(1) $$F_{Lmi}=\frac{N_{Lmi}}{\overset{n_{L}}{\underset{i=1}{\sum }}NLmi}$$

(2) $$F_{\mathrm{GCm}j}=\frac{N_{\mathrm{GCm}j}}{\overset{n_{\mathrm{GC}}}{\underset{j=1}{\sum }}N_{\mathrm{GCm}j}}$$

(3) $$F\mathrm{mat}k=\frac{N_{\mathrm{mat}k}}{\overset{n_{\mathrm{mat}}}{\underset{k=1}{\sum }}N_{\mathrm{mat}k}}$$

Where, *F*_*L*m*i*_ is the frequency of the optimal matched segments whose length are within the *i*th group, *N*_*L*m*i*_ is the amount of the optimal matched segments in the *i*th group, and *n*_*L*_ is the amount of the groups divided according to their lengths. *F*_GCm*j*_ is the frequency of the optimal matched segments whose GC contents are within the *j*th group, *N*_GCm*j*_ is the amount of the optimal matched segments in the *j*th group, and *n*_GC_ is the amount of the groups divided according to their GC contents. *F*_mat*k*_ is the frequency of the optimal matched segments whose matching rate are within the *k*th group, *N*_mat*k*_ is the amount of the optimal matched segments in the *k*th group, and *n*_mat_ is the amount of the groups divided according to their matching rates.

The lengths of the first introns in different gene sequences are different, we standardized the first introns as sequences with 100 bp length in order to conveniently compare the relative position distributions of the optimal matched segments. The method of length standardization as follows (Zhang et al., 2016a),


(4) $$n_{ij}=\left\{\begin{array}{cc} \left[100*N_{ij}/L_{i}\right] & 100*N_{ij}/L_{i}isinteger\\ \left[100*N_{ij}/Li\right]+1 & 100*N_{ij}/L_{i}\;\;isnon‐integer \end{array}\right.$$

Where, *L_i_* is the length of the *i*th first intron, *N_ij_* is the *j*th base site of the *i*th first intron, and *n_ij_* is its relative position corresponding to the *i*th standardized first intron. In this way, the first introns are all transformed into 100 bp long sequences.

According to the base site of each optimal matching sequence on the first intron, each base site of the first intron is scored, if in the optimal matching region, base site is scored 1, but if not, it is scored 0, and the definition of matching score as follows (Zhang et al., 2016a),


(5) $$f_{ij}=\{\begin{array}{cc} 1 & n_{ia}\leq j\leq n_{ib}\\ 0 & j\langle n_{ia}\;\;or\;\;j\rangle n_{ib\;\;} \end{array}$$

Where, *f_ij_* is the score of the *j*th base site on the standardized *i*th first intron, *n_ia_* and *n_ib_* are the initiation base relative site and the termination base relative site of the optimal matched segments on the standardized *i*th first intron. Thus, for each optimal matching sequence, the first intron is transformed into a sequence consisted of 0 and 1. if there are *m* optimal matching sequences in a gene, we can obtain *m* sequences consisted of 0 and 1. On this basis, we divided the 100 sites of each number sequences into 10 regions on average, the relative position frequency of the optimal matched segments on each site and in each region are defined as follows,


(6) $$F_{rj}=\overset{m}{\underset{i=1}{\sum }}f_{ij}/\overset{m}{\underset{i=1}{\sum }}\left(N_{ib}-N_{ia}+1\right)$$

(7) $$F_{rk}=\overset{p_{kb}}{\underset{j=p_{ka}}{\sum }}\overset{m}{\underset{i=1}{\sum }}f_{ij}/\overset{m}{\underset{i=1}{\sum }}\left(N_{ib}-N_{ia}+1\right)$$

Where, *F*_r*j*_ is the relative position frequency of the optimal matched segments on the *j*th base site of the standardized first intron, *F*_r*k*_ is the relative position frequency of the optimal matched segments in the *k*th region, *f_ij_* is the score of *j*th base site on the standardized first intron, *p_ka_* and *p_kb_* are the initiation base site and the termination base site of the *k*th region, *N_ia_* and *N_ib_* are the initiation base site and the termination base site of the optimal matched segments, and *m* is the total number of the optimal matched segments in the gene.

## Results

**Figure 1:**
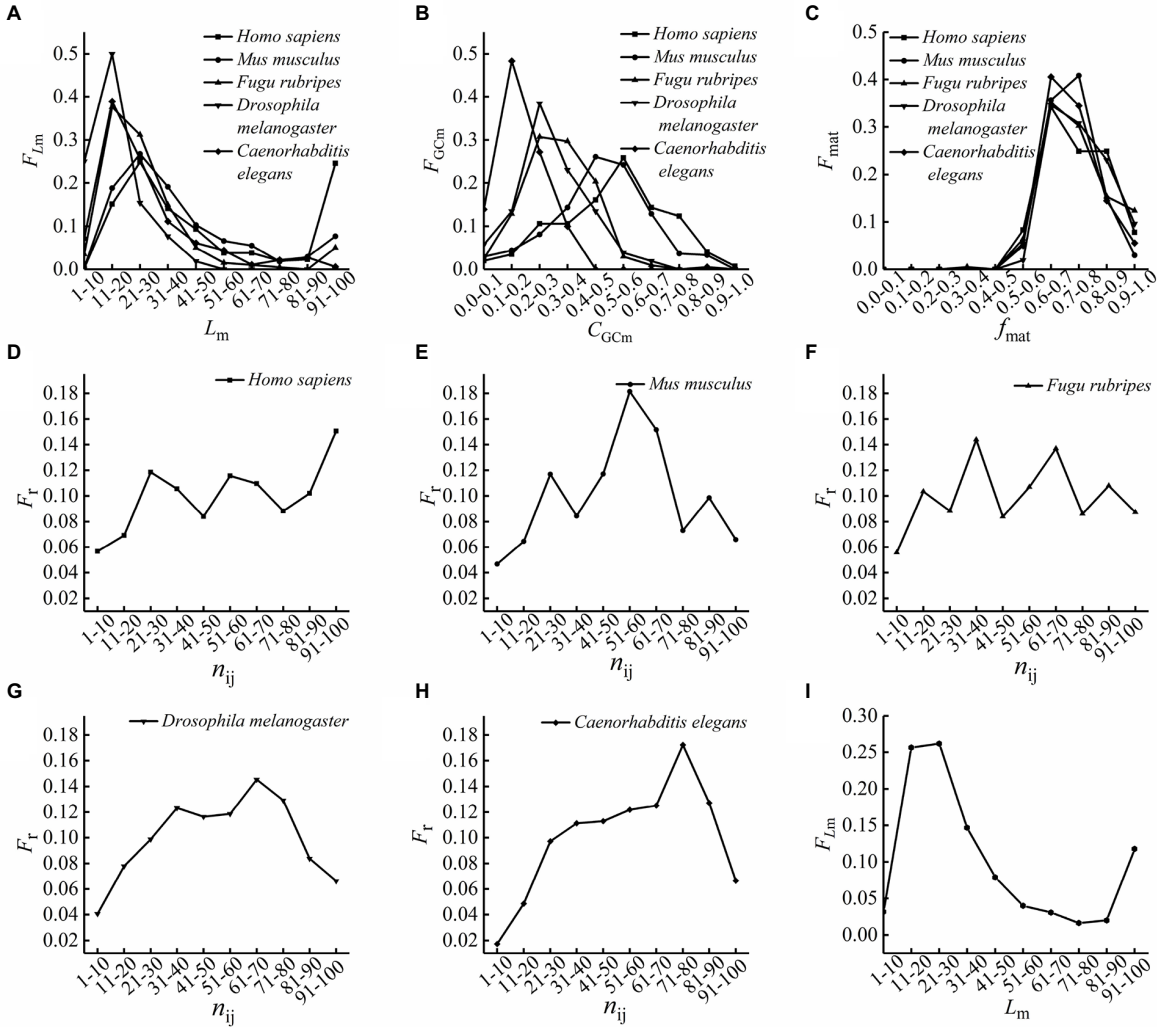
The distributions of the optimal matching frequencies. **(A)** The length (*L_m_*) distributions of the optimal matched segments. The values on the *x*-axis are the length ranges of groups divided according to lengths of the optimal matched segments, and those on the *y*-axis are the length frequency of the optimal matched segments. **(B)** The GC content (*C*_GCm_) distributions of the optimal matched segments. The values on the *x*-axis are GC content ranges of groups divided according to GC contents of the optimal matched segments, and those on the *y*-axis are the GC content frequencies of the optimal matched segments. **(C)** The matching rate (*f*_mat_) distributions of the optimal matched segment of five species. The values on the *x*-axis are matching rate ranges of groups divided according to matching rates of the optimal matched segments, and those on the *y*-axis are the matching rate frequencies of the optimal matched segments. **(D–H)** The relative position distributions of the optimal matched segments on the first intron sequences. The values on the *x*-axis are relative position ranges of groups divided according to relative positions of the optimal matched segments, and those on the *y*-axis are the relative position frequencies of the optimal matched segments. **(D)** *Homo sapiens*, **(E)** *Mus musculus*, **(F)** *Fugu rubripes*, **(G)** *Drosophila melanogaster*, **(H)** *Caenorhabditis elegans*. **(I)** The length (*L_m_*) distributions of the optimal matched segments of 5 species. The calculations of the optimal matched segment lengths of 5 species are combined into one, the optimal matching segments were divided into several groups according to their lengths, the frequency of the optimal matched segments in each group was calculated, the values on the *x*-axis are the length ranges of groups, and those on the *y*-axis are the length frequency of the optimal matched segments.

The optimal matched segments between the first introns and the reverse complementary sequences of other introns in each mitochondrial ribonucleo protein gene of five species were counted, and the dataset of the optimal matched segments was established. Then, the lengths of the optimal matched segments of each species were counted, and the frequencies of the optimal matched segments with different ranges of lengths were calculated by formula (1). The GC contents of the optimal matched segments of each species were counted, and the frequencies of the optimal matched segments with different ranges of GC contents were calculated by formula (2). The matching rates of the optimal matched segments of each species were calculated, and the frequencies of the optimal matched segments with different ranges of matching rates were calculated by formula (3). The first introns were standardized to sequences with 100 bp length, and their relative base positions were calculated according to formula (4), and then, according to the base sites of each optimal matching sequence on the first intron, each base site of the first intron is scored according to formula (5), based on this, the relative position frequency were calculated according to formula (6) and (7). On this basis, the characteristics of the optimal matched segments of five species were analyzed. The results are presented in Figure 1.

### The length distributions of the optimal matched segments

The lengths of the optimal matched segments of five species are mainly concentrated at 10–50 bp, while some optimal matched segments of *Homo sapiens, Mus musculus* and *Fugu rubripes* are up to 100 bp in length, and the ratio of the optimal matched segments of *Homo sapiens* concentrating at 90–100 bp is up to 24.6 percent. In addition, the length distribution of the optimal matched segments of *Homo sapiens* is similar to that of *Mus musculus*. The results showed that the optimal matched segments of high eukaryotes have a longer length and a wider length distribution than that of the low eukaryotes, and it means the length and the ranges of length distribution of the optimal matched segments are increased along with the evolution of eukaryotes.

### The GC content distributions of the optimal matched segments

The distributions of GC content of the optimal matched segments of five species ranged from 0 to 0.9. And comparing the results of the five species, it is found that the peak values of *F*_GCm_ are decreased along with the evolution of eukaryotes, but the corresponding GC content of the peak values are increased along with the evolution of eukaryotes.

### The matching rate distributions of the optimal matched segments

As seen from the matching rate distributions of the optimal matched segments, most of the matching rates are distributed between 60% and 80%. Interestingly, studies showed that the matching rates between miRNA and target mRNA are distributed between 65% and 95% (Cui et al., 2010). Comparing the matching rate ranges of the optimal matched segments with that of siRNA or miRNA with target mRNA (Volpe et al., 2002; Lim et al., 2005; Cui et al., 2010; Zhang et al., 2016b), it is found that there is a high similarity between the optimal matched segments with the most probable matching rates and siRNA or miRNA, this also suggests these optimal matched segments may be related to some non-coding RNAs with special biological functions, just like siRNA and miRNA, they may play an important role in the process of gene expression and regulation.

### The relative position distributions of the optimal matched segments in the first introns

It can be seen from Figure 1 that the relative positions of the optimal matched segments vary with the different species. And the peaks with *F*r values bigger than 10% are analyzed, the results showed that for *Homo sapiens*, there are 3 peaks with Fr values bigger than 10%, which are at 20–30 bp, 50–60 bp and 90–100 bp, and there is two peaks with Fr values bigger than 10%, which are at 20–30 bp and 50–60 bp for *Mus musculus*, 30–40 bp and 60–70 bp for *Fugu rubripes* and *Drosophila melanogaster*, but for *Caenorhabditis elegans*, there is only one peak with Fr value bigger than 10%, which is at 70–80 bp. This also indicates that the relative position frequencies of the optimal matched segments are distinctively differences among the five different species, and the peaks of relative position distributions of optimal matched segments are increased along with the evolution of eukaryotes, but the positions of the first two peaks exhibit significant conservatism.

**Figure 2:**
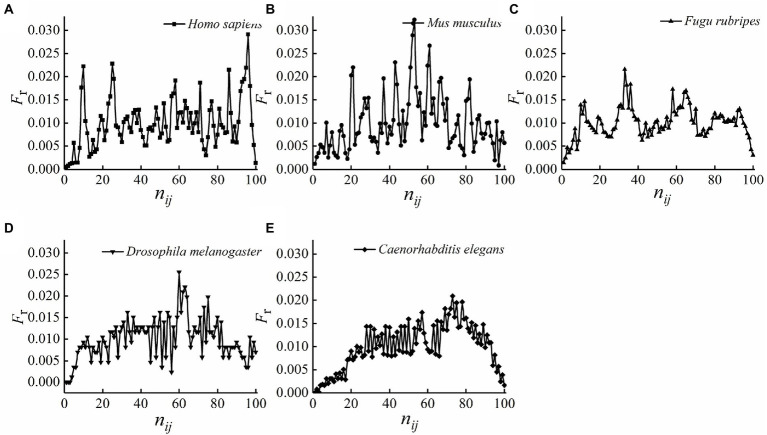
**(A–E)** The distribution of the relative position frequencies with the base sites of the standardized first intron.

In order to further confirm the conservatism of the relative position distributions of optimal matched segments, Figure 2 was made according to the calculations by formula (6), which express the relative position frequencies with the base sites of the standardized first intron.

As we can see from Figure 2, the distributions do not accord with normal distribution, so, for the relative position frequency of the optimal matched segments on each site of the standardized first intron, the test for differences between any two species were performed by non-parametric test with level of significance 0.05 using R software, the results are presented in Table 2.

**Table 2:** Results of the test for differences of relative position frequency of the optimal matched segments on each site of the standardized first intron.

Species	Value of *p*	Species	Value of *p*
*Homo sapiens-Mus musculus*	0.54	*Mus musculus-Drosophila melanogaster*	0.30
*Homo sapiens-Fugu rubripes*	0.40	*Mus musculus-Caenorhabditis elegans*	0.33
*Homo sapiens-Drosophila melanogaster*	0.66	*Fugu rubripes-Drosophila melanogaster*	0.91
*Homo sapiens-Caenorhabditis elegans*	0.52	*Fugu rubripes-Caenorhabditis elegans*	0.68
*Mus musculus-Fugu rubripes*	0.10	*Drosophila melanogaster-Caenorhabditis elegans*	0.64

It can be seen from Table 2 that the *p*-values between any two species are >0.05, it indicates that the differences between any two species were not statistically significant, means, in terms of the distributions on each site of the standardized first intron, the optimal matched segments exhibited high conservatism during species evolution. These results further confirmed the conservatism of the relative position distributions of optimal matched segments, which let us be sure that optimal matched segments are organized and functional sequences for each species.

## Conclusion

We analyzed the matching features between the first intron and the reverse complementary sequences of other introns in each gene sequence, for the lengths of the optimal matched segments, we found the most probable lengths are distributed between 20 and 30 bp, it can be seen from Figure 1, and we calculated the ratios of the optimal matched segments whose lengths are between 21 and 30 bp, they are 25%, 27%, 31%, 15%, and 26% in *Homo sapiens*, *Mus musculus*, *Fugu rubripes*, *Drosophila melanogaster* and *Caenorhabditis elegans, respectively.* Interestingly, we know that the siRNA, whose length is from 21 to 25 bp, guiding mRNA to silent by perfect complementarity with target mRNA (Volpe et al., 2002; Lim et al., 2005), and the miRNA, whose length is from 18 to 25 bp, restrains transcription and expression of target mRNA by different degree complementarity with target mRNA (Zhang et al., 2016a), the results indicate that the probable lengths of the optimal matched segments are remarkably similar to the lengths of siRNA and miRNA. For the matching rates of the optimal matched segments, we found that most of the matching rates are distributed between 60% and 80%, they are very remarkably similar to the matching rate ranges with target mRNA of siRNA or miRNA. It means there is a high similarity between some optimal matched segments and siRNA or miRNA. Is this a coincidence? we do not think so. The basic interaction between introns is base complementary pairing, the matched sequences, especially between the first intron and corresponding complementary sequences of other introns in the same gene, must be related to some elements with special functions. Taking all the analyzes and conclusions above into account, we come to a conclusion that some optimal matched segments may be a kind of non-coding RNA with special biological functions, just like siRNA and miRNA, they are likely to participate in the process of gene expression and regulation. And we think, the optimal matched segments with special characteristics in the first introns may take part in regulating gene expression by RNA matching competition with other introns or exon.

In addition, we have got some interesting results by comparing the results of different species. In terms of the species selected in this work, *Caenorhabditis elegans*, *Drosophila melanogaster*, *Fugu rubripes*, *Mus musculus*, and *Homo sapiens* are listed from lower eukaryotes to higher eukaryotes. Based on this order and the calculations, we tried to analyze the variation law of matching features of introns along with the species evolution. For the lengths of the optimal matched segments, the average length of the optimal matched segments for the high eukaryotes are longer than that of the low eukaryotes. It suggests that the lengths of the optimal matched segments are increased in the evolution of eukaryotes. And the results showed that with the evolution of eukaryotes, the distributions of the length of the optimal matched segment become wider and wider. It means the lengths and the ranges of length distributions of the optimal matched segments are increased along with the evolution of eukaryotes. For the GC content of the optimal matched segments, the peak values of *F*_GCm_ are decreased along with the evolution of eukaryotes, it suggests the GC contents of the optimal matched segments are more widely distributed with the evolution of eukaryotes. If some functional elements are related to the optimal matched segments with special GC contents, the result means that higher organisms have more kinds of functional elements than lower organisms. But the corresponding GC content at the peak values are increased along with the evolution of eukaryotes. Both AT and GC basepairs form one set of hydrogen bonds, and it is a truth universally acknowledged that a GC base pair has three hydrogen bonds whereas AT has two, it means that DNA with high GC-content is more stable than DNA with low GC-content. Based on the above theories, it can be concluded that introns of higher organisms interacting with each other though weak bonds binding are more than that of lower organisms, we hypothesized that interactions through weak bonds can ensure the flexibility to take part in gene regulation. For the relative position of the optimal matched segments, the peaks of relative position distributions of optimal matched segments are increased with the evolution of eukaryotes, and the positions of the first two peaks exhibit significant conservatism. We think that some functional elements are related to the optimal matched segments at these proper positions, the results indicated that these elements of higher eukaryotes may have a more specific division of labor.

To conclude, in this work, we analyzed the possibility of interactions between the first introns and the other introns of mitochondrial ribosomal protein genes, then tried to interpret the modes of interactions between introns. We found some universal characteristics of the optimal matched segments between the first introns and the reverse complementary sequences of other introns, and we noticed there is a high similarity between some optimal matched segments and siRNA or miRNA, so, we believe that the characteristics of interactions among introns obtained in this work are the basic characteristics of the RNA–RNA interactions. It means the optimal matched segments are probably functional non-coding RNAs including siRNA and miRNA. At the same time, we found some variation law of the optimal matched segments with the evolution of eukaryotes, which indicates that there may be a great difference in the complexity of the interactions of introns among species at different evolutionary levels, these results are of great significance in explaining the function of non-coding RNA. An increasing number of people are realizing the importance of non-coding RNA in gene expression regulating, but the functions and regulating mechanisms are not very clear, our results indicate that the base matching plays a key role in the interactions among introns. However, the related works have just started, the sample size in this study is relatively small, further large-sample studies are needed to obtain more detailed and clearer mechanism of introns interactions.

## Data availability statement

The original contributions presented in the study are included in the article/supplementary material, further inquiries can be directed to the corresponding author.

## Author contributions

RFL, XWS, SG, and SYP performed the work. RFL has developed the theoretical frame work, finished the data analysis, and wrote the manuscript. XWS, SG, and SYP finished the data collection and the calculations. All authors have read and approved this version of the article.

## Funding

This work was supported by the Natural Science Foundation of Inner Mongolia (2019MS03042) and the National Natural Science Foundation of China (31860304).

## Conflict of interest

The authors declare that the research was conducted in the absence of any commercial or financial relationships that could be construed as a potential conflict of interest.

## Publisher’s note

All claims expressed in this article are solely those of the authors and do not necessarily represent those of their affiliated organizations, or those of the publisher, the editors and the reviewers. Any product that may be evaluated in this article, or claim that may be made by its manufacturer, is not guaranteed or endorsed by the publisher.

## Footnotes

^1^ http://mobyle.pasteur.fr/cgi-bin
